# Inhibition of the MyD88 signaling pathway could upregulates Ghrelin expression to synergistically regulate hepatic *Echinococcus multilocularis*-infected progression

**DOI:** 10.3389/fimmu.2024.1512180

**Published:** 2024-12-19

**Authors:** Jiang Zhu, Tanfang Zhou, Guangfeng Chen, Yuhui Wu, Xia Chen, Ya Song, Ayinula Tuohetali, Huijing Gao, Dongming Pang, Hao Wen, Kalibixiati Aimulajiang

**Affiliations:** ^1^ Department of Abdominal Surgery, The Third People Hospital of Xinjiang Uygur Autonomous Region, Urumqi, China; ^2^ State Key Laboratory of Pathogenesis, Prevention and Treatment of High Incidence Diseases in Central Asia, Clinical Medicine Institute, The First Affiliated Hospital of Xinjiang Medical University, Urumqi, China; ^3^ The First Affiliated Hospital, School of Medicine, Shihezi University, Shihezi, China; ^4^ College of Veterinary Medicine, Xinjiang Agricultural University, Urumqi, China

**Keywords:** MyD88, Ghrelin, alveolar echinococcosis, *Echinococcus multilocularis*, hepatic

## Abstract

**Introduction:**

AE and whether the inhibition of the MyD88 inflammatory pathway can enhance Ghrelin expression to collaboratively modulate AE progression remains unclear.

**Methods:**

In this study, we evaluated Ghrelin serum levels and changes in TLR4/MyD88/NF-κB pathway proteins and inflammatory factors in AE patients and *E. multilocularis* mouse models at different stages of infection (-4, -8, and -12 weeks). Additionally, we administered the MyD88 inhibitor TJ-M2010-5 intraperitoneally to infected mice to evaluate alterations in inflammation and Ghrelin levels, as well as disease progression.

**Results:**

A decrease in serum Ghrelin levels in AE patients, whereas both Ghrelin and GHSR, along with TLR4/MyD88/NF-κB pathway proteins and markers of M1/M2 macrophage polarization, exhibited increased expression in the inflammatory cell zones surrounding hepatic lesions. Similar findings were observed in *E. multilocularis*-infected mice. M1-type inflammatory expression predominated throughout the infection’s progression, with sustained high levels of Ghrelin counteracting inflammation. The TLR4/ MyD88/NF-κB pathway remained suppressed during the first 8 weeks, becoming activated only at 12 weeks. Inhibition of the MyD88 pathway resulted in reduced inflammation levels and upregulated Ghrelin expression, thereby collaboratively regulating the progression of hepatic infection.

**Conclusion:**

These findings suggest an interactive regulation between the MyD88 inflammatory signaling pathway and Ghrelin, indicating that MyD88 inhibition could enhance Ghrelin expression to modulate the progression of *E. multilocularis* infection.

## Introduction

Alveolar echinococcosis (AE) is a severe zoonotic disease caused by Echinococcus multilocularis (*E. multilocularis*) infection, leading to the formation of malignant tumor-like lesions in the liver. The parasitic growth of *E. multiloculari* is typically slow and often difficult to detect in the early stages. Over time, it can progress to extensive, infiltrative lesions, with surgical intervention remaining the primary treatment; without timely excision, the mortality rate exceeds 90% (1–3). Currently, AE is frequently diagnosed at advanced stages, where significant vascular and biliary invasion complicates surgical resection. Additionally, the immune mechanisms underlying AE pathogenesis remain poorly understood, and effective immunotherapeutic strategies are still under investigation. Therefore, continued research into the immune inflammatory and growth metabolic regulatory mechanisms of AE is essential to identify potential immunotherapeutic targets or small molecular compounds against this disease.

MyD88 is a pivotal protein that links pathogen and damage signals to cellular immune responses. It interacts with various pattern recognition receptors (PRRs), including Toll-like receptors (TLRs) and the interleukin-1 receptor (IL-1R) family, thereby activating inflammatory signaling through the Toll/IL-1 receptor (TIR) domain. Upon activation, MyD88 initiates downstream signaling pathways, such as NF-κB and mitogen-activated protein kinase (MAPK), which lead to the release of inflammatory cytokines and the onset of inflammatory responses (4–7). Research on MyD88 in liver diseases has highlighted its crucial role in regulating hepatic inflammation and metabolism. Recent studies indicate that MyD88 in myofibroblasts enhances the development of nonalcoholic fatty liver disease (MASLD)-related liver cancer by promoting M2 macrophage polarization (8). Although MyD88 is essential for modulating liver inflammation, its overexpression can result in pathological conditions such as inflammatory damage, autoimmune diseases, and transplant rejection (9–11). In the context of hepatic echinococcosis, the MyD88 signaling pathway also exerts a regulatory role. An experiment involving partial hepatectomy in mice demonstrated that *E. multilocularis* infection significantly increased serum MyD88 levels, potentially inhibiting liver regeneration (12). Furthermore, studies have shown that MyD88 expression levels steadily increase over time in mice infected with *E. granulosus (* 13). Chronic infection with AE may activate the MyD88 signaling pathway, which could counteract parasite invasion while potentially mediating host tissue damage. TJ-M2010-5, a novel MyD88 inhibitor, effectively suppresses inflammatory responses by blocking the TLR/MyD88 signaling pathway (14, 15). Preclinical studies have demonstrated its protective effects against various inflammatory diseases. For instance, in models of inflammation-related colorectal cancer, TJ-M2010-5 can inhibit intestinal inflammation, thereby delaying tumor onset and progression (16). In models of acute liver injury, it alleviates inflammatory damage to the liver and provides organ protection (17). Additionally, in allogeneic transplantation models, TJ-M2010-5 can mitigate immune cell attacks on transplant tissues, reducing rejection responses (18). While research suggests that blocking the MyD88 signaling pathway may slow the pathological progression of inflammation-related diseases, the specific molecular mechanisms remain to be elucidated.

Ghrelin, an endogenous ligand for the growth hormone secretagogue receptor (GHSR), was first identified in the stomachs of rats and humans in 1999 (19). As a gastric peptide, Ghrelin plays a vital role not only in regulating appetite and energy balance but also in maintaining liver health. Moreover, the Ghrelin-IGF-1 growth axis is a critical pathway for regulating systemic growth metabolism, immune inflammation, glucose-lipid homeostasis, and autophagic apoptosis, functioning as a classic route within the “gastrointestinal-brain-liver axis” to modulate hepatic metabolic activities (20–22). Ghrelin has been demonstrated to inhibit the MyD88/NF-κB inflammatory signaling pathway (23–26) and the TGF-β/Smad3 fibrotic signaling pathway (27, 28), while also promoting hepatic regeneration and repair. This provides protective effects against immune inflammation and fibrogenesis in conditions such as nonalcoholic fatty liver disease (MASLD), hepatitis, liver fibrosis, cirrhosis, and liver injury. Recent studies further indicate that IGF-1, regulated by Ghrelin, may facilitate the progression of various parasitic diseases, potentially linked to improved immune inflammatory microenvironments that promote immune tolerance and evasion, thereby aiding parasite growth (29–33). Our research group has identified an IGF-1 signaling pathway in Echinococcus larvae, with blocking IGF-1 receptors resulting in the collapse of hepatic echinococcosis lesions and mitigation of disease progression. While several studies suggest an interactive regulatory relationship between growth metabolism and immune inflammation pathways, the specific involvement of Ghrelin in the modulation of alveolar echinococcosis (AE) and whether inhibition of the MyD88 inflammatory pathway can upregulate its expression to collaboratively regulate AE progression remain unclear.

In this study, we comprehensively investigate the involvement of Ghrelin and the TLR4/MyD88/NF-κB pathway in regulating the disease progression of *E. multilocularis* infection in the liver. We aim to determine whether the inhibition of the MyD88 signaling pathway can upregulate Ghrelin expression and collaboratively modulate the progression of hepatic *E. multilocularis* infection. The results are anticipated to reveal new targets for immunotherapy against alveolar echinococcosis (AE) from the perspective of growth metabolic pathways.

## Materials and methods

### Clinical sample

This retrospective study included a cohort of healthy individuals and confirmed alveolar echinococcosis (AE) patients who underwent surgical treatment at the First Affiliated Hospital of Xinjiang Medical University between September 2022 and September 2023. Healthy individuals were classified into the healthy control (HC) group (n = 20), while AE patients were categorized into the AE group (n = 20). The classification of AE was based on the revised criteria established by the World Health Organization Informal Working Group on Echinococcosis (WHO-IWGE) (34, 35). Serum samples were collected from both groups, along with liver samples from the AE group, which comprised infected lesions and adjacent liver tissue (CLT, defined as “proximal liver tissue,” approximately 0.5 cm from the lesion) (36).

### Animals

This study utilized female C57BL/6J and BALB/c mice, aged 8 to 10 weeks and weighing 20 ± 2 grams, obtained from the Medical Experimental Animal Center of Xinjiang Medical University (Xinjiang, China). The mice were housed in a specific pathogen-free environment at the Animal Experiment Center of Xinjiang Medical University, maintained under controlled temperature and humidity with a 12-hour light/dark cycle, and had ad libitum access to food and water. Prior to the experiments, the mice underwent a fasting period of 6 hours.

### Mouse model of *E. multilocularis* infection

Protoscoleces (PSCs) were injected into the peritoneal cavity of gerbils, with euthanasia performed approximately 10 weeks post-infection. Under sterile conditions, cyst fluid and vesicular tissue were collected, and the PSCs were harvested through grinding, washing, and filtering. The PSCs were then digested in a 1% pepsin solution at 37°C for 30 minutes, with the pH adjusted to 3.0 (37). Their viability was assessed using eosin staining, with a viability rate of >95% deemed suitable for mouse modeling.

The mouse experiments consisted of two parts. In the first part, C57BL/6 mice were randomly assigned to six groups based on *E. multilocularis* infection status and duration of rearing: Control and *E.m* groups for 4, 8, and 12 weeks (6 mice/group). Mice undergo abdominal surgery under 1% isoflurane anesthesia. the Control group received an equivalent volume of saline via the portal vein, while the *E.m* group was injected with 0.1 mL of saline containing 4000 PSCs via the portal vein (36, 38). After the specified breeding period, euthanize the mice using the physical method of cervical dislocation, and serum and liver tissue samples were collected. In the second part, BALB/c mice were randomly divided into three groups based on *E. multilocularis* infection status and drug intervention: Control, *E.m*, and *E.m* + TJ-M2010-5 groups (6 mice/group). Following inhalation anesthesia, laparotomy was performed; the Control group received an equivalent volume of saline via the portal vein, while the *E.m* group was injected with 0.1 mL of saline containing 2000 PSCs via the portal vein. The *E.m* + TJ-M2010-5 group began intraperitoneal injections of TJ-M2010-5 four weeks post-*E. multilocularis* modeling, with injections administered for six consecutive days followed by one day of rest. At week 12, all three groups of mice were euthanized using isoflurane anesthesia and cervical dislocation, and serum and liver tissue samples were collected.

### Western blot

Total protein was extracted from mouse liver and quantified using a BCA protein assay kit (Solarbio, Beijing, China). Protein separation was performed using SDS-PAGE gel (Biotides, Beijing, China), followed by transfer onto a PVDF membrane (Sigma-Aldrich, Shanghai, China). The membrane was incubated overnight at 4°C with primary antibodies diluted in a blocking solution containing 5% non-fat milk. The following day, after washing, the membrane was incubated with an HRP-conjugated secondary antibody (multi-rAb HRP-goat anti-rabbit, 1:5000, RGAR001, Proteintech, Wuhan, China). Enhanced ECL reagent (Biosharp, Hefei, China) was utilized for the detection of target proteins. The primary antibodies used included: anti-rabbit MyD88 (1:1000, AF5195); anti-rabbit GHSR (1:1000, AF2794, Affinity, Ohio, USA); anti-rabbit iNOS (1:1000, ab283655); anti-rabbit Arg-1 (1:1000, ab315110); anti-rabbit NF-κB p65 (1:1000, ab16502, Abcam, Cambridge, UK) and anti-rabbit TLR4 (1:1000, 26156-1-AP, Proteintech, Wuhan, China). Anti-rabbit β-actin (1:5000, 20536-1-AP, Proteintech, Wuhan, China) was used as a loading control for normalization.

### Enzyme-linked immunosorbent assay

Quantification of human serum Ghrelin and IGF-1 levels was performed using an ELISA kit and a microplate reader set to a wavelength of 450 nm (BioTek, Vermont, USA). Additionally, serum levels of Ghrelin, GHSR, IGF-1, IGF-1R, ALT, AST, monoamine oxidase (MAO), and prolyl hydroxylase (PH) were measured in mice. Furthermore, liver tissue levels of Ghrelin, GHSR, IGF-1, IGF-1R, IL-2, TNF-α, IFN-γ, IL-4, IL-10, and NF-κB p65 were assessed. All ELISA kits were obtained from Lapuda Biotechnology (Nanjing, China), except for the PH kit, which was sourced from Jin Yibai Biotechnology (Nanjing, China). The ELISA procedures were conducted following the manufacturer’s instructions.

### Quantitative real-time polymerase chain reaction

Total RNA from mouse liver was extracted using Trizol reagent, followed by quantification of RNA concentration. The extracted mRNA was reverse transcribed into cDNA using the HiScript III RT SuperMix for qPCR kit (Vazyme, Nanjing, China). Quantitative real-time PCR (qRT-PCR) reactions were performed with the SYBR Green I detection kit (Vazyme, Nanjing, China), and fluorescence signals were monitored using a Bio-Rad real-time PCR system (Bio-Rad, Foster City, CA). Specific forward and reverse primers for qRT-PCR were designed and synthesized by Sangon Biotech (Shanghai, China). The primers used were as follows: Ghrelin forward primer 5’-GCACCAGAAAGCCCAGAGAAAG-3’ and reverse primer 5’-TCTTCTGCTTGTCCTCTGTCCTC-3’; GHSR forward primer 5’-ACCGTGATGGTATGGGTGTCG-3’ and reverse primer 5’-CACAGTGAGGCAGAAGACCG-3’; β-actin forward primer 5’-GGGACGACATGGAGAGAG-3’ and reverse primer 5’-ACGACCAGAGGCATACAG-3’.

### Immunohistochemical staining

Liver tissues from AE patients and mice were fixed in 4% formaldehyde solution (Biosharp, Hefei, China), embedded in paraffin, and sectioned to a thickness of 4 μm. The sections were dewaxed and rehydrated using a gradient of ethanol to remove xylene. Antigen retrieval was performed according to the manufacturer’s instructions using Tris-EDTA and citrate buffer, followed by blocking endogenous peroxidase activity with 3% H_2_O_2_. Goat serum was applied for blocking for 30 minutes (Proteintech, Wuhan, China). Primary antibodies were incubated overnight at 4°C. The following day, secondary antibody incubation was conducted using goat anti-rabbit/mouse HRP-conjugated polymer (Proteintech, Wuhan, China), and color development was performed with the DAB kit (Proteintech, Wuhan, China), followed by counterstaining with hematoxylin solution (ZSGB-Bio, Beijing, China). Observations were made using an optical microscope (Olympus, Tokyo, Japan), and images were captured and analyzed for cell positivity rates using Image-J software. The primary antibodies used included: anti-rabbit Ghrelin (1:7000, ab20979); anti-rabbit NF-κB p65 (1:1000, ab16502); anti-rabbit CD68 (1:1000, ab303565); anti-rabbit iNOS (1:100, ab115819, Abcam, Cambridge, UK); anti-rabbit TLR4 (1:100, AF7017); anti-rabbit GHSR (1:300, DF2794, Affinity, Ohio, USA); anti-rabbit GHRL (1:400, 13309-1-AP); anti-rabbit Arg-1 (1:200, 16001-1-AP); anti-rabbit MyD88 (1:200, 67969-1-Ig); anti-rabbit IGF-1 (1:200, 20215-1-AP) and anti-rabbit IGF-1R (1:2400, 20254-1-AP, Proteintech, Wuhan, China).

### Hematoxylin and eosin staining and Masson’s trichrome staining

First, mouse liver specimens were fixed in 4% formaldehyde solution (Biosharp, Hefei, China) to preserve tissue structure integrity. Following fixation, the specimens underwent processing for embedding, resulting in paraffin sections for subsequent staining and microscopic observation. The sections were stained with hematoxylin-eosin (H&E) and Masson’s trichrome staining (Solarbio, Beijing, China). Stained sections were observed under an optical microscope to assess structural and pathological changes in liver tissue. Images captured using an optical microscope (Olympus, Tokyo, Japan) were recorded for further analysis and documentation.

### Fluorescence-based multiplex immunohistochemistry staining

Liver sections from mice were first dewaxed in xylene and subsequently rehydrated through a series of ethanol solutions. Antigen retrieval was performed using citrate buffer, followed by blocking with a serum working solution. The sections were then incubated overnight with primary antibodies at 4°C. The next day, they were treated with corresponding HRP-conjugated secondary antibodies for 50 minutes, followed by tyramide signal amplification with a fluorescent dye in the dark for 10 minutes. Microwave retrieval was performed, and this process was repeated for additional primary antibodies. Finally, DAPI (Servicebio, Wuhan, China) was used to stain the nuclei, and the sections were cover slipped and imaged using a fluorescence microscope (Nikon, Tokyo, Japan). The primary antibodies employed in this study included: anti-rabbit Ghrelin (1:5000, ab20979; Abcam, Cambridge, MA, USA), anti-rabbit CD3 (1:1000, GB12014), anti-rabbit CD68 (1:3000, GB113109), anti-rabbit MyD88 (1:3000, GB111554) and anti-rabbit NF-κB (1:1000, GB11997; Servicebio, Wuhan, China).

### Statistical analysis

In this study, statistical analyses were conducted using SPSS v26.0, and graphical representations of the results were generated with GraphPad Prism 9.0. Statistical results are typically presented as mean ± standard deviation, while categorical variables are expressed as frequency or percentage. To evaluate significant differences between groups, we employed t-tests or one-way ANOVA, based on results from at least three independent experiments. Correlation analysis was performed using the Spearman correlation coefficient. Data were deemed statistically significant at: * *p* < 0.05; ** *p* < 0.01; *** *p* < 0.001; **** *p* < 0.0001.

## Results

### Ghrelin and TLR4/MyD88/NF-κB signaling pathways in the development of AE liver lesions

**Figure 1:**
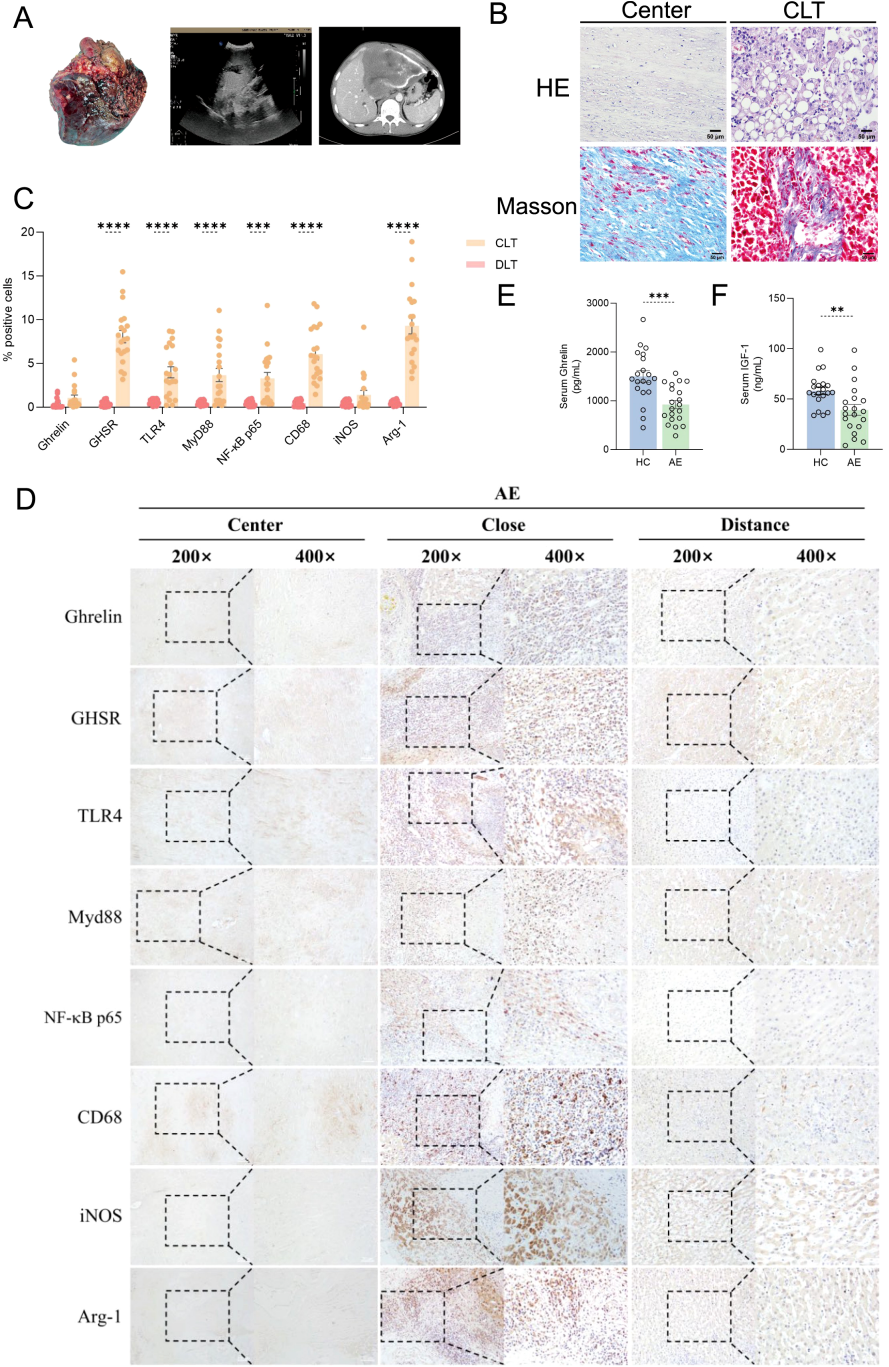
Ghrelin and TLR4/MyD88/NF-κB signaling pathways in the development of AE liver lesions. **(A)** Imaging studies, including ultrasound and CT scans, of liver resection specimens from AE patients. **(B)** H&E and Masson staining of liver sections from AE patients. **(C, D)** IHC staining analysis of protein expression levels of Ghrelin, GHSR, TLR4, MyD88, NF-κB p65, CD68, iNOS, and Arg-1 in the livers of AE patients, along with quantification of the percentage of positive cells. Histological images were captured at magnifications of 200x and 400x. CLT indicates "close" liver tissue. **(E, F)** Quantitative detection of Ghrelin and IGF-1 levels in serum samples from the HC and AE groups via ELISA. Statistics are presented as mean ± SD. **p < 0.01, ***p < 0.001, and ****p < 0.0001.

We initially assessed the size of lesions in AE patients using ultrasound and CT imaging, confirming that all liver lesions were active (**Figure 1A**). General demographic data for both groups, including gender and age, are presented in **Table 1**, while clinical data regarding lesion size, location, and PNM-p staging for the AE group are detailed in **Table 2**. Histological examination via H&E and Masson staining revealed that the centers of the lesions predominantly consisted of solid blue collagen fibers, with vacuoles or granular substances evident in the cytoplasm of hepatocytes at the lesion edges (CLT), alongside extensive infiltration of inflammatory cells and scattered blue collagen fibers. This observation indicates disruption of the liver lobular structure and proliferation of fibrous tissue (**Figure 1B**). Immunohistochemical (IHC) staining results demonstrated that the centers of AE liver lesions were nearly completely calcified, exhibiting rare cellular components, while a wide band of inflammatory cells surrounding the lesions expressed Ghrelin and its receptor GHSR (**Figure 1D**). Furthermore, abundant expression of TLR4/MyD88/NF-κB pathway proteins and macrophages was observed in the inflammatory cell band (**Figure 1C**). Analysis of the percentage of positive cells revealed a significant increase compared to liver tissue distally from the lesions. Notably, protein expressions of Ghrelin, GHSR, TLR4, MyD88, and NF-κB p65 were localized to the peripheral region of the inflammatory cell band, indicating activation of growth metabolism and involvement of immune inflammation in the progression of liver lesions. Interestingly, macrophage polarization status revealed that activation of the M1 polarization marker iNOS mainly occurred in the peripheral part of the inflammatory cell band, while the M2 polarization marker Arg-1 was primarily activated in the inner and central regions. These findings suggest that Ghrelin may regulate M1 polarization of macrophages via the TLR4/MyD88/NF-κB signaling pathways, thereby balancing inflammation and tissue repair in the development of AE liver lesions. Additionally, serum levels of Ghrelin (**Figure 1E**) and IGF-1 (**Figure 1F**) were measured via ELISA, showing significantly lower levels in AE patients compared to the HC group. These results indicate that systemic expression of the Ghrelin-IGF-1 growth axis is altered in the terminal stage of AE, potentially relating to disease progression and serving as a protective mechanism to limit parasitic disease advancement.

**Table 1:** The general data of the AE group.

Characteristics		n (%)
Total patients included		20 (100)
Sex	Male Female	11 (55) 9 (45)
Age (y)	20-40 40-60	5 (25) 15 (75)
Cystic lesion location (CT)	Left lobe of liver Right lobe of liver Whole liver	4 (20) 13 (65) 3 (15)
Cystic lesion size (CT)	≦10cm >10cm	8 (40) 12 (60)
PNM-p	2 3	17 (85) 3 (15)

**Table 2:** The general data of the control group and AE group.

Characteristics	Male n(%)	Age (y)
Control group(n=20)	11 (55)	50.40 ± 9.78
AE group (n=20)	10 (50)	46.45 ± 16.59
t/x^2^	0.1003, 1	0.9323
P	0.7515	0.3629

### As *E. multilocularis* infection progresses in mice, liver levels of Ghrelin significantly increase

**Figure 2:**
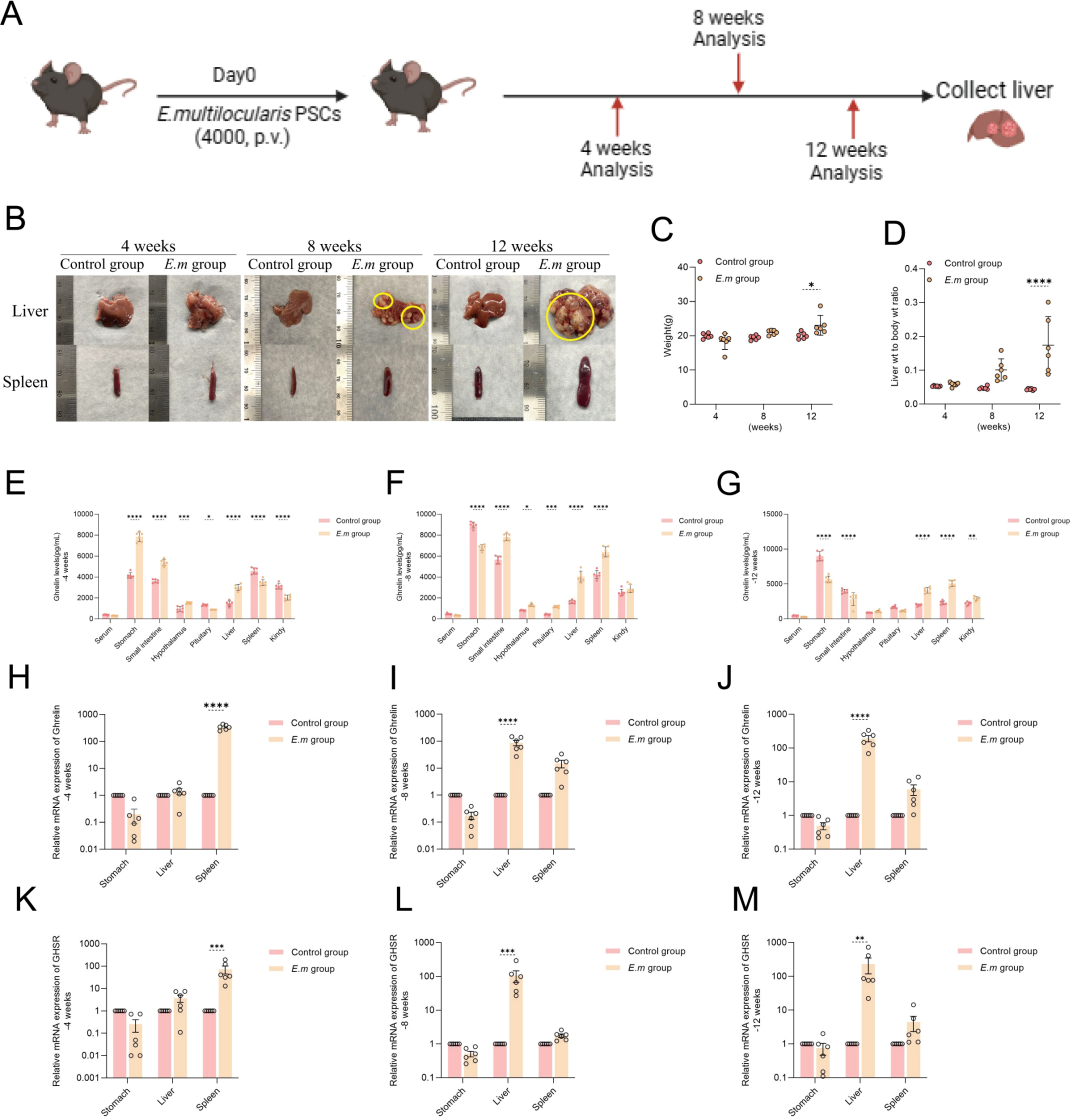
As *E*. *multilocularis* infection progresses in mice, liver levels of Ghrelin significantly increase. **(A)** Establishment of *E*. *multilocularis* infection mouse models at different time points (-4, -8, and -12 weeks). **(B)** Following model establishment, liver and spleen tissues were collected for morphological observation to assess macroscopic changes during the infection process. **(C)** Weight changes of infected mice at different time points were recorded to evaluate the impact of infection on growth. **(D)** Changes in the liver-to-body weight ratio of infected mice were monitored to understand the infection's effect on the liver. **(E–G)** Ghrelin levels in serum, stomach, small intestine, hypothalamus, pituitary, liver, spleen, and kidneys at various time points in *E*. *multilocularis*-infected mice were measured via ELISA to explore the impact of infection on Ghrelin distribution. **(H–J)** The relative expression levels of Ghrelin mRNA in the stomach, liver, and spleen of infected mice at different time points were assessed by qRT-PCR to evaluate the effect of infection on Ghrelin gene expression. **(K–M)** The relative expression levels of GHSR mRNA in the stomach, liver, and spleen of *E*. *multilocularis*-infected mice were also measured by qRT-PCR to elucidate how infection affects GHSR gene expression. Statistics are presented as mean ± SD. *p< 0.05, **p < 0.01, ***p < 0.001, and ****p < 0.0001.

To investigate the variations in Ghrelin levels at different stages of *E. multilocularis* infection, we established liver infection models and observed them at 4-, 8-, and 12-weeks post-infection (**Figure 2A**). Morphological changes in the liver and spleen were recorded through macroscopic observation (**Figure 2B**), and we meticulously tracked changes in body weight at different time points (**Figure 2C**), alongside measuring liver weight to calculate the liver-to-body weight ratio (**Figure 2D**). At 12 weeks, we observed a significant increase in both body weight and liver-to-body weight ratio in the model group compared to the control group, reflecting the rapid progression of hepatic infection from weeks 8 to 12. We utilized ELISA to measure Ghrelin levels in serum and various organs at different time points. At 4 weeks, Ghrelin levels in the stomach, small intestine, hypothalamus, and liver of the model group were significantly higher than those in the control group (**Figure 2E**). This suggests that during the early stage of *E. multilocularis* infection, the host attempts to increase Ghrelin secretion by activating the “gastrointestinal-brain-liver axis,” possibly to boost energy reserves against infection while also inhibiting immune inflammation and promoting liver repair to support parasite survival and development. Conversely, Ghrelin levels in the pituitary, spleen, and kidneys were significantly lower than in the control group, indicating that *E. multilocularis* infection may influence the distribution and regulation of Ghrelin within the body. At 8 weeks, Ghrelin levels in the stomach of the model group were significantly lower than in the control group (**Figure 2F**), whereas levels in the small intestine, hypothalamus, pituitary, liver, and spleen were significantly higher, reflecting changes in Ghrelin demand by immune-regulating organs during the infection. By 12 weeks, the decrease in Ghrelin levels in the stomach and small intestine became more pronounced (**Figure 2G**), yet the liver and spleen—key organs in regulating immune inflammation during parasitic liver infection—remained significantly elevated. Additionally, we assessed the expression levels of Ghrelin and GHSR genes in the stomach, liver, and spleen via qRT-PCR (**Figures 2H–J**). The results mirrored the trends observed in the ELISA data (**Figures 2K–M**), where Ghrelin and GHSR gene expression in the stomach of the model group consistently remained lower than in the control group, while levels in the liver and spleen were consistently elevated. These data demonstrate that during the progression of *E. multilocularis* infection, the regulatory activities of immune organs, particularly the liver and spleen, necessitate substantial recruitment of Ghrelin, potentially inhibiting gastrointestinal Ghrelin secretion through a negative feedback mechanism of the “gastrointestinal-brain-liver axis.” This suggests a regulatory role of Ghrelin on organ function and inflammatory response throughout the infection process.

### Ghrelin and the TLR4/MyD88/NF-κB signaling pathway participate in regulating the development of *E. multilocularis* infection in liver lesions

**Figure 3:**
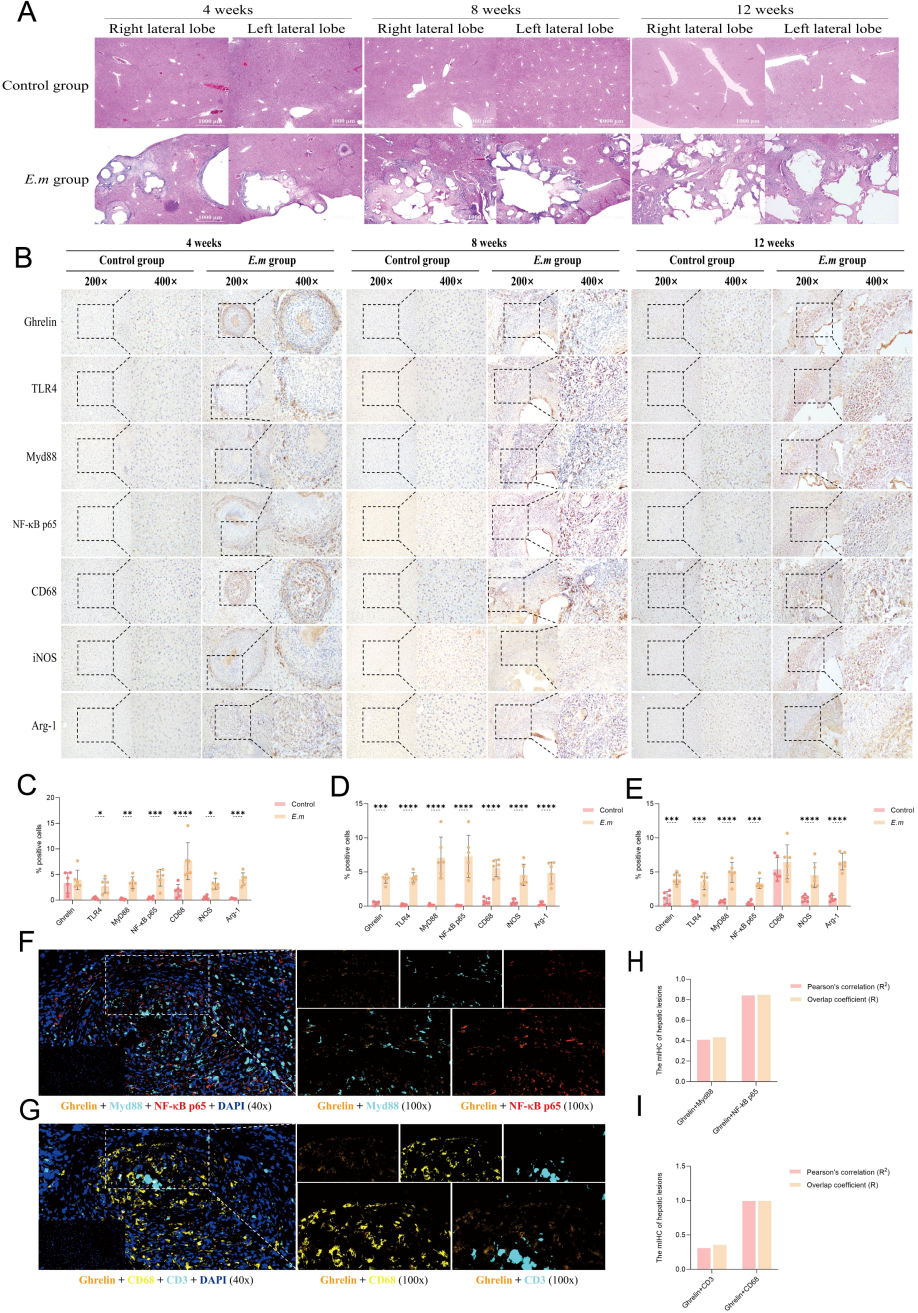
Ghrelin and the TLR4/MyD88/NF-κB signaling pathway participate in regulating the development of *E*. *multilocularis* infection in liver lesions. **(A)** H&E staining was performed on liver tissues from infected mice at various stages to observe and assess pathological changes. **(B)** IHC staining was conducted to detect the protein expression of Ghrelin, TLR4, MyD88, NF-κB p65, CD68, iNOS, and Arg-1 in the liver, noting the primary expression locations. **(C–E)** Quantitative analysis of the percentage of positive cells from the aforementioned IHC images to assess the impact of infection on protein expression. Histological images were captured at magnifications of 200x and 400x. **(F)** mIHC was utilized to observe immunofluorescence co-staining images of DAPI (blue), Ghrelin (orange), MyD88 (cyan), and NF-κB p65 (red) in liver tissue sections from infected mice, revealing the intracellular distribution and interactions of these proteins. **(G)** mIHC also examined the co-staining images of DAPI (blue), Ghrelin (orange), CD68 (yellow), and CD3 (cyan) to investigate the co-localization of Ghrelin with immune cell markers. **(H)** We calculated the Pearson correlation coefficient and overlap coefficient between Ghrelin and MyD88, NF-κB p65 to analyze their co-localization within cells. **(I)** Similarly, we calculated the Pearson correlation coefficient and overlap coefficient between Ghrelin and CD68, CD3 to assess co-localization with these immune cell markers. Statistics are presented as mean ± SD. *p < 0.05, **p < 0.01, ***p < 0.001, and ****p < 0.0001.

In this study, we successfully replicated the IHC findings from alveolar AE patients by observing liver *E. multilocularis* infection at different stages of progression in mice. We first performed H&E staining to assess changes in tissue structure (**Figure 3A**). With prolonged infection duration, we observed a significant increase and enlargement of liver lesions, particularly during the rapid progression from weeks 8 to 12. These lesions primarily manifested as multiple cyst formations, surrounded by a dense band of inflammatory cells, invading and infiltrating normal liver tissue without clear boundaries. The inner side of the inflammatory cell band was characterized mainly by calcification. IHC staining results demonstrated abundant protein expression of Ghrelin, TLR4/MyD88/NF-κB pathway proteins, and macrophages in the inflammatory cell band surrounding the liver lesions at all stages of disease progression (**Figure 3B**). Analysis of the percentage of positive cells (**Figures 3C–E**) revealed a significant increase compared to the control group. Moreover, the protein expressions of Ghrelin, TLR4, MyD88, and NF-κB p65 were consistently localized to the peripheral part of the inflammatory cell band. Similar patterns were observed in macrophage polarization markers, where activation of the M1 marker iNOS predominantly occurred in the peripheral region, while activation of the M2 marker Arg-1 was primarily found in the inner and central areas. These data suggest that Ghrelin may play a role in the development of E. multilocularis liver lesions by regulating the polarization state of macrophages through the TLR4/MyD88/NF-κB signaling pathway. To visually demonstrate the correlation between Ghrelin and various cells and pathway proteins, we employed multiplex immunohistochemistry (mIHC) to observe the co-localization of Ghrelin with MyD88, NF-κB p65, CD68, and CD3 (**Figures 3F, G**). The results indicated strong co-localization of Ghrelin with NF-κB p65 and CD68, as well as with MyD88 and CD3 (**Figures 3H, I**). These findings further support our conclusion that Ghrelin is involved in regulating macrophage polarization mediated by the MyD88/NF-κB signaling pathway.

### *E. multilocularis* infection activates the TLR4/MyD88/NF-κB signaling pathway and upregulates M1 macrophage polarization mediating pro-inflammatory responses

**Figure 4:**
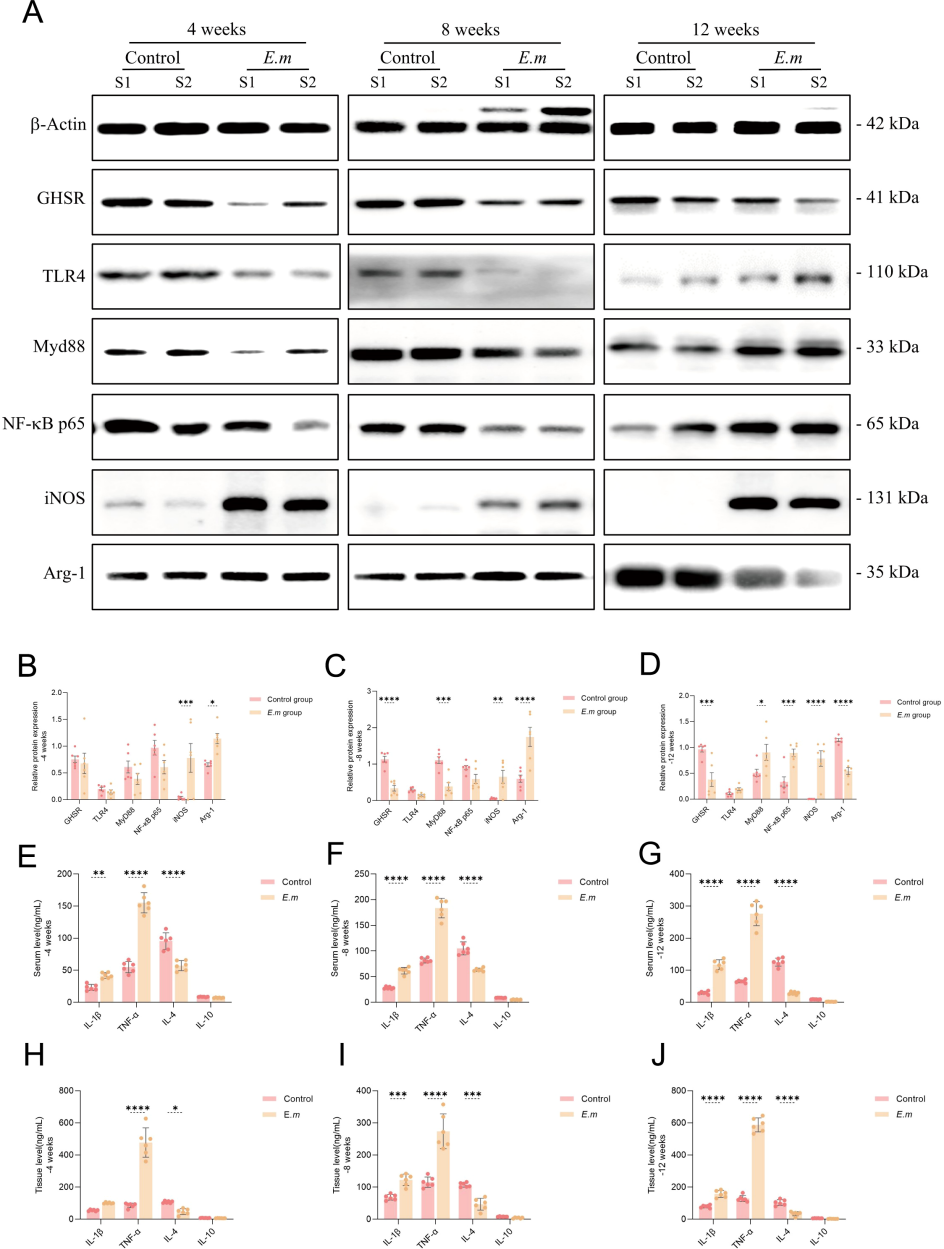
*E. multilocularis* infection activates the TLR4/MyD88/NF-κB signaling pathway and upregulates M1 macrophage polarization mediating pro-inflammatory responses. **(A)** WB analysis was conducted to detect the expression levels of GHSR, TLR4, MyD88, NF-κB p65, iNOS, and Arg-1 proteins in the liver of infected mice at different stages to evaluate changes in these key proteins. **(B–D)** Statistical analysis of the relative expression levels of proteins from the aforementioned WB results included three independent experiments to ensure data reliability and reproducibility. **(E–G)** Additionally, we measured the levels of M1 polarization secretory factors (IL-1β and TNF-α) and M2 polarization secretory factors (IL-4 and IL-10) in the serum of infected mice at various stages via ELISA to assess the systemic immune response. **(H–J)** We also conducted ELISA to assess the aforementioned immune factor levels in liver tissue to analyze the local immune response in infected mice. Statistics are presented as mean ± SD. *p < 0.05, **p < 0.01, ***p < 0.001, and ****p < 0.0001.

To investigate the changes in the TLR4/MyD88/NF-κB signaling pathway and macrophage polarization states during various stages of *E. multilocularis* infection in mice, we conducted a series of quantitative protein analyses on liver tissues (**Figure 4A**). The results indicated that GHSR protein levels continuously decreased throughout the infection (**Figures 4B–D**), highlighting a negative correlation between Ghrelin and total liver receptor protein levels, consistent with previous studies (39). During the initial 8 weeks, the activity of the TLR4/MyD88/NF-κB signaling pathway decreased; however, a significant increase was observed by week 12. This pathway plays a crucial role in regulating immune inflammation, and its early decline suggests a suppression of inflammatory responses during the initial phase of infection, potentially facilitating immune evasion by the parasite. Conversely, the subsequent increase in activity may indicate ongoing infection or inflammation, enhancing the activation of inflammatory signaling pathways or reflecting the host’s attempt to boost the inflammatory response to eliminate the pathogen. Throughout the first 8 weeks, liver protein levels of the M1 macrophage marker iNOS and the M2 macrophage marker Arg-1 significantly increased (**Figures 4B, C**), suggesting a synergistic role for both macrophage types during the initial infection phase. By week 12, while iNOS levels continued to rise alongside TLR4/MyD88/NF-κB pathway activation, Arg-1 levels decreased (**Figure 4D**). This disruption in M1/M2 polarization balance indicates a shift towards a predominant pro-inflammatory response aimed at combating the parasitic infection. We further assessed changes in serum levels of M1 polarization secretory factors (including IL-1β and TNF-α) and M2 factors (including IL-4 and IL-10) using ELISA (**Figures 4E–G**), as well as liver levels (**Figures 4H–J**). The results demonstrated that, influenced by the liver parasitic infection, pro-inflammatory factors IL-1β and TNF-α exhibited continuous increases in both serum and liver levels at all infection stages, while anti-inflammatory factors IL-4 and IL-10 showed persistent decreases. These findings suggest that prior to TLR4/MyD88/NF-κB pathway activation, M1 macrophage-mediated inflammatory factors had already begun to be secreted to facilitate immune clearance of the parasitic infection.

### Inhibition of MyD88 signaling pathway exacerbates the progression of liver lesions in *E. multilocularis* infection

**Figure 5:**
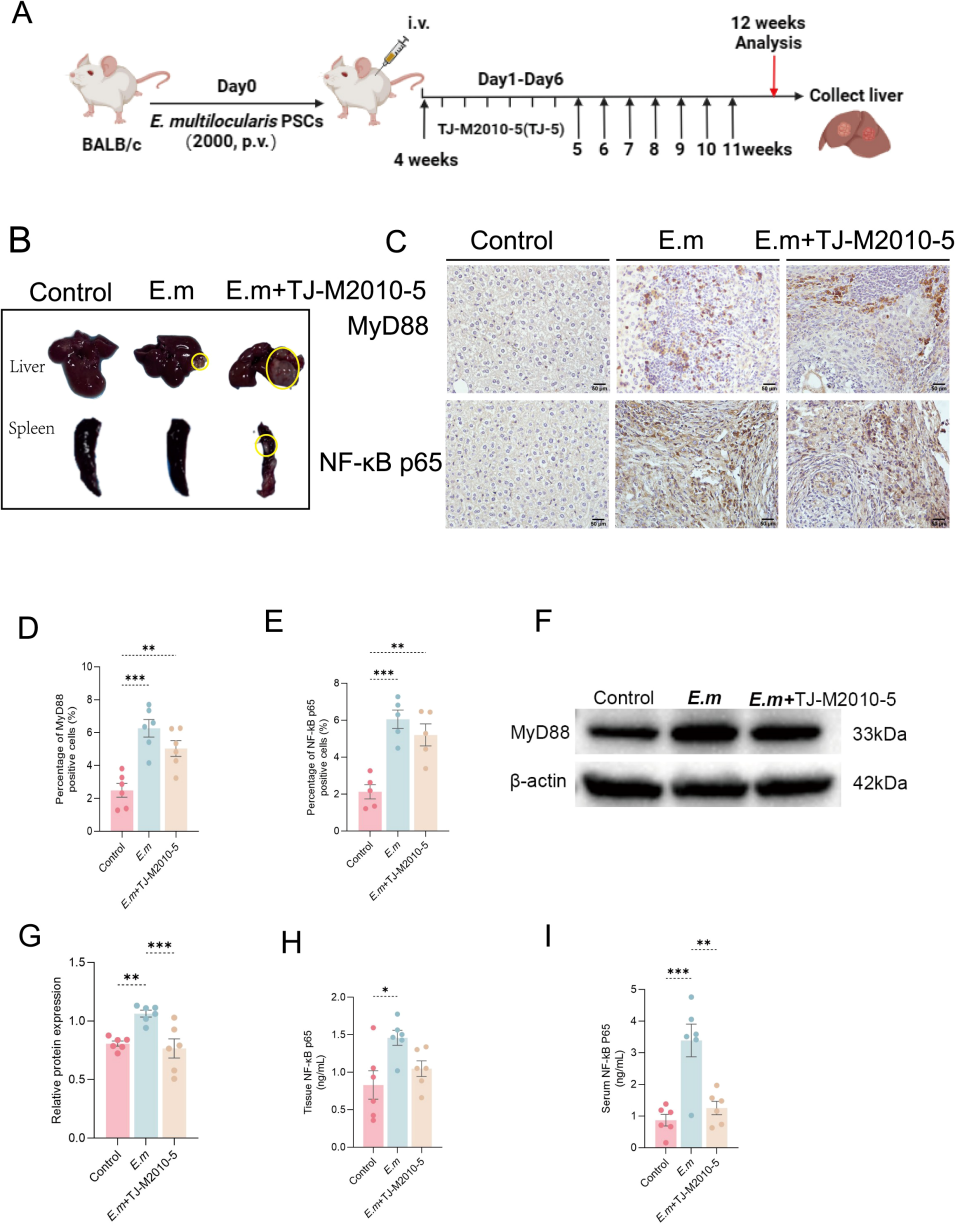
Inhibition of MyD88 signaling pathway exacerbates the progression of liver lesions in *E*. *multilocularis* infection. **(A)** We established a mouse model of *E*. *multilocularis* infection and administered a MyD88 blocker via intraperitoneal injection according to the predetermined protocol. **(B)** Morphological observations of all mouse livers and spleens were conducted to evaluate macroscopic changes in the organs. **(C)** IHC staining was performed on liver tissue sections to evaluate the expression changes of MyD88 and NF-κB p65 in response to infection and intervention. **(D, E)** The percentage of positive staining area in IHC images was calculated to assess the proportion of positive cells for MyD88 and NF-κB p65. **(F)** MyD88 expression levels in liver proteins among the three groups of mice were detected using WB analysis. **(G)** Statistical analysis of the relative expression levels of liver tissue proteins included three independent repeat experiments to ensure reliability and reproducibility of the results. **(H, I)** ELISA measured NF-κB p65 levels in serum and liver tissue to evaluate the impact of intervention on downstream pathway proteins. Statistics are presented as mean ± SD. *p < 0.05, **p < 0.01, ***p < 0.001, and ****p < 0.0001.

Research has demonstrated that suppression of immune inflammation can facilitate immune evasion by parasites, worsening parasitic infections (40). Given that TLR4 inhibitors can lead to rapid mortality in mice (8, 41), we chose the MyD88 blocker TJ-M2010-5 to intervene in *E. multilocularis*-infected mice (**Figure 5A**). This intervention aimed to determine whether inhibiting the MyD88 signaling pathway could upregulate Ghrelin expression and collaboratively influence the progression of liver infection. Through macroscopic observation of liver and spleen changes (**Figure 5B**), we found that MyD88 pathway inhibition resulted in rapid progression of liver infection lesions, potentially accumulating in the spleen. Analysis of liver tissue section immunohistochemistry (IHC) staining (**Figure 5C**) revealed significantly higher protein expression of MyD88 (**Figure 5D**) and NF-κB p65 (**Figure 5E**) in the inflammatory cell band of liver lesions in both the *E.m* and *E.m* + TJ-M2010-5 groups compared to the Control group, though the *E.m* + TJ-M2010-5 group exhibited lower levels than the *E.m* group. Quantitative analysis of liver MyD88 protein levels via Western blot (**Figure 5F**) confirmed that the *E.m* group was significantly higher than both the Control and E.m + TJ-M2010-5 groups (**Figure 5G**). Furthermore, serum and liver homogenate ELISA (**Figures 5H, I**) indicated that systemic and liver levels of NF-κB p65 mirrored the trends observed with MyD88, with the *E.m* group showing higher levels than the Control and *E.m* + TJ-M2010-5 groups. These data confirm that the MyD88 inhibitor TJ-M2010-5 effectively suppresses the MyD88/NF-κB inflammatory signaling pathway.

### Inhibition of MyD88 signaling pathway upregulates Ghrelin expression and reduces pro-inflammatory response during *E. multilocularis* infection

**Figure 6:**
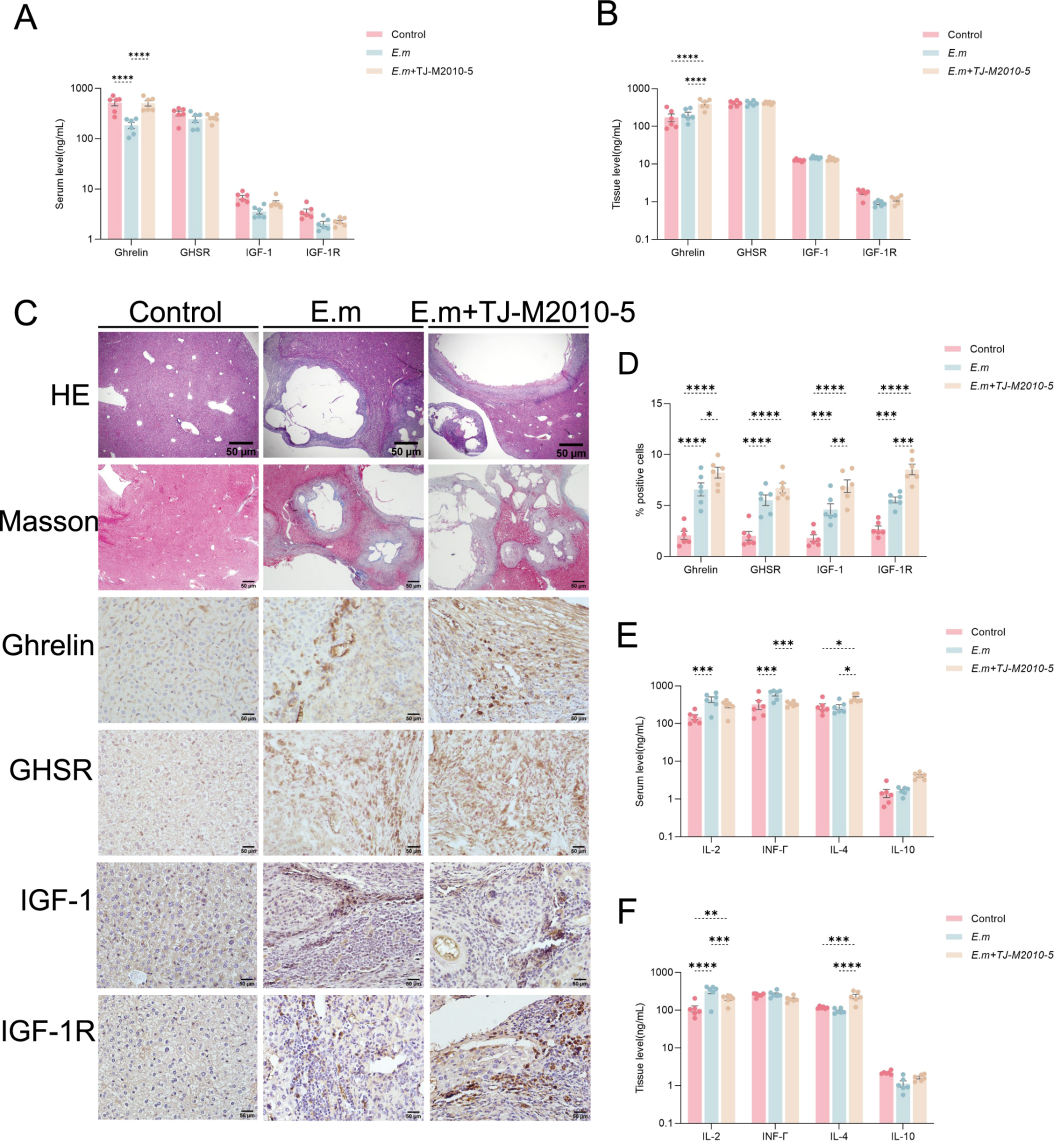
Inhibition of MyD88 signaling pathway upregulates Ghrelin expression and reduces pro-inflammatory response during *E*. *multilocularis* infection. **(A, B)** ELISA was used to detect the levels of Ghrelin, GHSR, IGF-1, and IGF-1R in serum and liver tissues to evaluate their expression changes at systemic and local levels. **(C)** H&E and Masson staining were performed on liver tissue sections from infected mice to examine changes in pathological morphology and collagen fiber extent, along with IHC staining to assess the protein expression of Ghrelin, GHSR, IGF-1, and IGF-1R. **(D)** The percentage of positive staining area for Ghrelin, GHSR, IGF-1, and IGF-1R was calculated. **(E, F)** Additionally, we detected the levels of pro-inflammatory cytokines (including IL-2 and INF-γ) and anti-inflammatory cytokines (including IL-4 and IL-10) in serum and liver tissues via ELISA to analyze the immune status of infected mice. Statistics are presented as mean ± SD. *p < 0.05, **p < 0.01, ***p < 0.001, and ****p < 0.0001.

In this study, we further investigated whether suppression of the MyD88 signaling pathway could activate the Ghrelin-IGF-1 growth axis. ELISA results from serum and liver tissue homogenates (**Figures 6A, B**) indicated a significant increase in Ghrelin levels in the *E.m* + TJ-M2010-5 group compared to the *E.m* group, while levels of GHSR, IGF-1, and IGF-1R showed no significant changes. Additionally, we performed H&E staining, Masson staining, and immunohistochemistry (IHC) on liver tissue sections (**Figure 6C**). Both H&E and Masson staining results demonstrated that infection lesions were significantly more severe in the *E.m* + TJ-M2010-5 group compared to the *E.m* group. IHC analysis (**Figure 6D**) revealed that the number of positive cells expressing Ghrelin, GHSR, IGF-1, and IGF-1R in the inflammatory cell band of liver lesions was significantly higher in the *E.m* group compared to the Control group. However, the number of positive cells expressing Ghrelin, IGF-1, and IGF-1R in the *E.m* + TJ-M2010-5 group was significantly greater than in both previous groups. These data indicate that inhibiting the MyD88 signaling pathway can upregulate Ghrelin expression at both systemic and local levels during the progression of *E. multilocularis* infection. Ghrelin has been shown to suppress immune inflammation in the liver (39, 42). ELISA results (**Figures 6E, F**) demonstrated that pro-inflammatory cytokines IL-2 and IFN-γ were significantly elevated in the *E.m* group compared to the *E.m* + TJ-M2010-5 group, while the level of the anti-inflammatory cytokine IL-4 was significantly higher in the *E.m* + TJ-M2010-5 group than in the other two groups.

The changes in the immune inflammatory microenvironment may be associated with high Ghrelin expression suppressing liver immune inflammation but could also result from the blockade of the MyD88 signaling pathway. In conclusion, the suppression of immune inflammation can lead to the progression of liver lesions in *E. multilocularis* infection.

### Inhibition of MyD88 signaling pathway mitigates liver fibrosis during *E. multilocularis* infection

**Figure 7:**
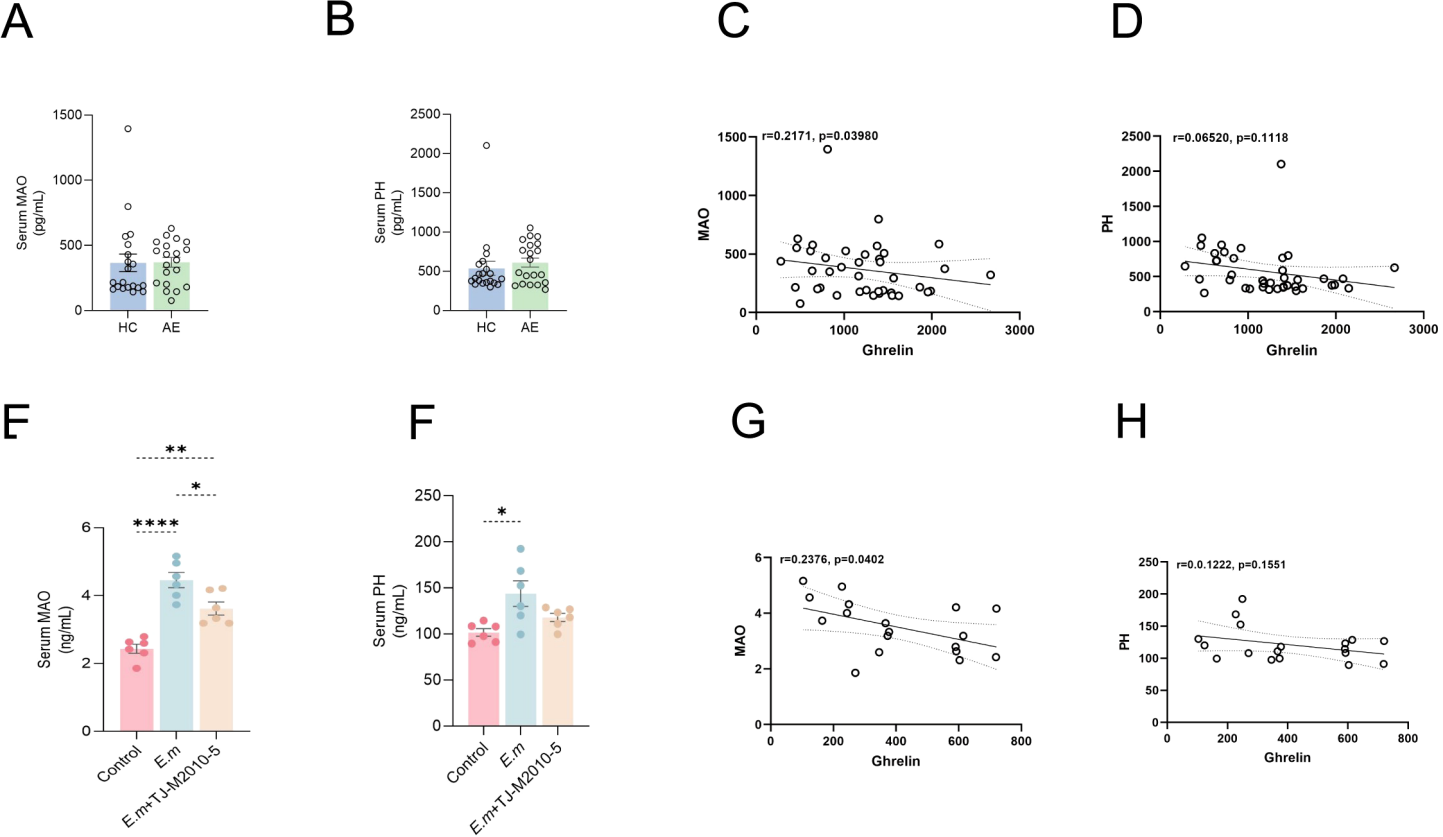
Inhibition of MyD88 signaling pathway mitigates liver fibrosis during *E*. *multilocularis* infection. **(A, B)** ELISA was performed to quantify MAO and pH levels in serum samples from the HC and AE groups. **(C, D)** Correlation analysis explored the relationship between Ghrelin and MAO, pH levels in AE patients. **(E, F)** In the drug intervention experiment using the *E. multilocularis* infection mouse model, serum MAO and pH levels were also measured. **(G, H)** The correlation between serum Ghrelin and MAO and PH levels in mice was analyzed. Statistics are presented as mean ± SD. *p < 0.05, **p< 0.01, ***p < 0.001, and ****p < 0.0001.

In this study, we also investigated changes in liver fibrosis markers, specifically MAO and pH levels, to assess overall liver fibrosis alterations. There were no significant differences in serum MAO and pH levels between the HC and AE groups (**Figures 7A, B**). Correlation analysis revealed a significant negative relationship between Ghrelin and MAO (**Figure 7C**), while the correlation with pH was not significant (**Figure 7D**).

To validate these findings, we conducted similar measurements in mouse models. ELISA results showed that serum MAO (**Figure 7E**) and pH (**Figure 7F**) levels were significantly elevated in the *E.m* group compared to the Control group; however, inhibition of the MyD88 signaling pathway resulted in a notable reduction in these levels. Furthermore, a significant negative correlation between Ghrelin and MAO was observed in the mouse model (**Figure 7G**), while the correlation with pH remained insignificant (**Figure 7H**). These findings suggest an interactive regulatory role between immune inflammatory pathways and fibrosis pathways (43–46). Ghrelin appears to ameliorate liver fibrosis formation (28), indicating that inhibiting the MyD88 signaling pathway to upregulate Ghrelin expression may collaboratively reduce the occurrence of liver fibrosis during *E. multilocularis* infection.

## Discussion

AE is a chronic parasitic infection that can lead to persistent inflammatory damage, fibrosis, and organ dysfunction in the liver (38, 47). When the immune inflammatory microenvironment in the liver is suppressed, it may result in immune tolerance and escape by the parasite, exacerbating disease progression (38, 48–50). Additionally, Ghrelin is known to inhibit pro-inflammatory responses, potentially influencing the progression of AE (23, 24, 26). Our findings indicate that at various stages of the disease in both AE patients and *E. multilocularis*-infected mice, there is significant protein expression of Ghrelin, TLR4/MyD88/NF-κB signaling pathways, and macrophages in the periphery of liver lesions. Immunohistochemical (IHC) staining clearly shows that Ghrelin, TLR4, MyD88, NF-κB p65, and the M1 polarization marker iNOS are expressed in the inflammatory cell band surrounding the lesions. Multiplex immunohistochemistry (mIHC) results further confirm strong co-localization of Ghrelin with NF-κB p65 and CD68, as well as MyD88. These data suggest that Ghrelin may regulate M1 macrophage polarization mediated by the TLR4/MyD88/NF-κB signaling pathway, balancing the inflammatory response and tissue repair induced by parasitic infection. Notably, while serum Ghrelin levels consistently decrease at different stages of AE, our investigation of various organs in *E. multilocularis*-infected mice revealed significantly elevated Ghrelin levels in the stomach, small intestine, hypothalamus, and liver at the four-week mark post-infection. In the early stages of *E. multilocularis* infection, the host may attempt to counter the infection by activating the “gut-brain-liver axis” to increase Ghrelin secretion, potentially enhancing energy reserves to fight the infection and playing a role in suppressing immune inflammation and promoting liver repair to assist parasite survival and development. As the infection progresses, Ghrelin levels in the stomach and small intestine begin to decline, while the liver and spleen—key organs in regulating immune inflammation—continue to show significantly elevated Ghrelin levels. These observations suggest that during the progression of *E. multilocularis* infection, the regulatory activities of the immune organs, such as the liver and spleen, require substantial recruitment of Ghrelin. This may involve a negative feedback mechanism to suppress Ghrelin secretion from the gastrointestinal tract via the “gut-hypothalamus-liver axis.” Furthermore, Ghrelin may modulate organ function and inflammatory responses through this classic pathway during parasitic infection.

The activation of the TLR4/MyD88/NF-κB signaling pathway has been shown to promote the secretion of immune factors and enhance dendritic cell (DC) antigen presentation, playing a crucial role in combating infectious diseases (13). MyD88, as a central mediator in this pathway, directly or indirectly influences the secretion of various downstream immune factors (51, 52). In the context of *E. multilocularis* infection, we observed that the TLR4/MyD88/NF-κB signaling pathway remains suppressed in the early stages of infection; however, it becomes activated after eight weeks. This early reduction in activity likely reflects an inhibition of the inflammatory response, facilitating immune evasion by the parasite and allowing it to survive and establish itself in the host. Conversely, the later activation of this pathway may indicate ongoing infection or inflammation, exacerbating inflammatory signaling. Throughout the infection, we noted that pro-inflammatory factors such as IL-1β and TNF-α consistently increased in both serum and liver tissue, while anti-inflammatory factors like IL-4 and IL-10 showed a continuous decline. These findings suggest that even before the activation of the TLR4/MyD88/NF-κB signaling pathway, M1 macrophage-mediated pro-inflammatory factors are already being secreted to facilitate the immune clearance of the parasitic infection. This interplay highlights the dynamic response of the immune system to *E. multilocularis* infection and the critical role of the TLR4/MyD88/NF-κB pathway in regulating inflammatory responses during disease progression.

In our study, we aimed to inhibit the MyD88 signaling pathway during the progression of *E. multilocularis* infection to determine whether this could upregulate Ghrelin expression and modulate disease progression. The results indicated that suppressing the MyD88 signaling pathway significantly upregulated Ghrelin expression both systemically and locally. Ghrelin appears to play a role in suppressing hepatic immune inflammation (39), which may synergistically reduce the host’s pro-inflammatory response, thereby affecting immune recognition and clearance of the parasite. This mechanism could contribute to the progression of liver lesions caused by *E. multilocularis* infection. Furthermore, our findings suggest an interaction between immune inflammatory pathways and fibrosis pathways (43–46), with Ghrelin potentially exerting a beneficial effect on reducing liver fibrosis formation (28). Inhibiting the MyD88 signaling pathway and upregulating Ghrelin expression might collaboratively mitigate the development of liver fibrosis during *E. multilocularis* infection. This could influence the formation of fibrous bands surrounding liver lesions, leading to a loss of self-limiting capacity and promoting rapid lesion growth. These insights underscore the complex interplay between immune regulation and fibrotic processes in the context of parasitic infections, emphasizing the potential therapeutic implications of targeting the MyD88 pathway and modulating Ghrelin levels.

**Figure 8:**
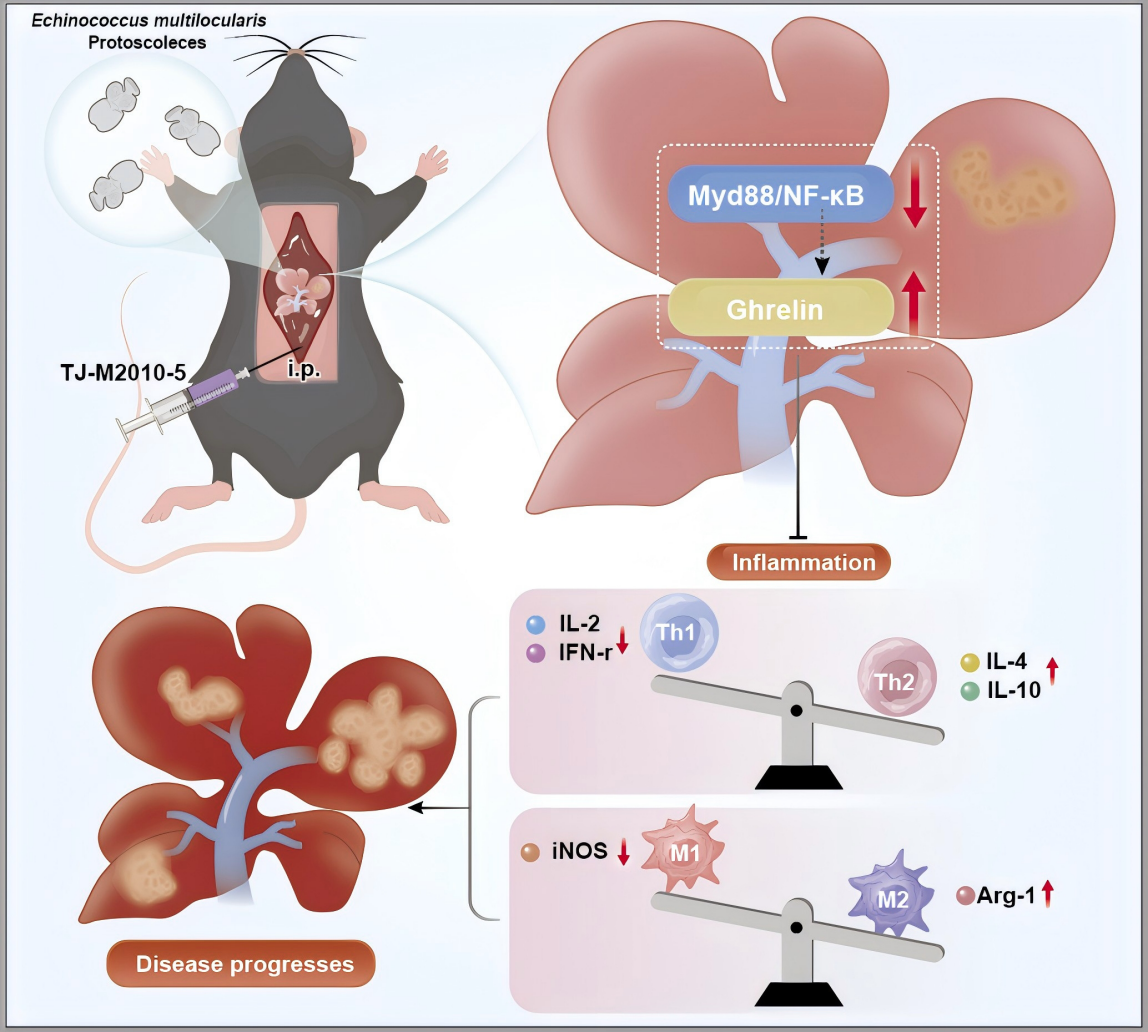
Inhibition of MyD88 signaling pathway can up-regulate Ghrelin expression and aggravate *Echinococcus multilocularis* disease progression. The picture showed that following the hepatic portal injection of *Echinococcus multilocularis* protoscoleces, the intraperitoneal administration of TJ-M2010-5 resulted in a marked suppression of the MyD88/NF-κB signaling pathway, accompanied by a significant increase in Ghrelin levels. These two factors synergistically mitigated the inflammatory response and modulated the imbalance between Th1 and Th2 cells. Specifically, the secretion of anti-inflammatory cytokines IL-4 and IL-10 was upregulated, while the secretion of pro-inflammatory cytokines IL-2 and INF-γ was downregulated. Furthermore, the expression of the M2 polarization factor Arg-1 was upregulated, whereas the expression of the M1 polarization factor iNOS was downregulated. These alterations may potentially exacerbate the progression of liver disease caused by *Echinococcus multilocularis* infection. The author used Adobe Illustrator to hand draw the images, and the original images had no copyright dispute.

This study reveals the involvement of Ghrelin and the TLR4/MyD88/NF-κB inflammatory signaling pathway in regulating the progression of *E. multilocularis* infection. Inhibiting the MyD88 signaling pathway was found to upregulate Ghrelin expression, collaboratively modulating disease progression in liver infections caused by *E. multilocularis*. The results suggest an interactive regulatory role between the MyD88 inflammatory signaling pathway and Ghrelin; however, the exact molecular mechanisms remain to be elucidated. For instance, understanding how the suppression of Ghrelin expression during *E. multilocularis* infection influences the behavior and function of immune cells, as well as its impact on disease progression, warrants further investigation (**Figure 8**). This research holds promise for exploring new therapeutic targets for AE immunotherapy through the lens of growth metabolic pathways. Future studies could provide valuable insights into the interplay between metabolic signaling and immune response in the context of parasitic infections.

## Data Availability

The raw data supporting the conclusions of this article will be made available by the authors, without undue reservation.
